# Self-Assessment of Knowledge on the Treatment of Children and Adolescents with Special Care Needs: Results of a Survey amongst German Dentists with Key Expertise in Paediatric Dentistry

**DOI:** 10.3390/jpm12071173

**Published:** 2022-07-19

**Authors:** Peter Schmidt, Daniela Reis, Andreas G. Schulte, Oliver Fricke

**Affiliations:** 1 Department of Special Care Dentistry, Witten/Herdecke University, 58455 Witten, Germany; andreas.schulte@uni-wh.de; 2 Department of Child and Adolescent Psychiatry, Psychotherapy and Child Neurology, Gemeinschaftskrankenhaus Herdecke, 58313 Herdecke, Germany; d.reis@gemeinschaftskrankenhaus.de (D.R.); oliver.fricke@uni-wh.de (O.F.); 3 Department of Child and Adolescent Psychiatry, Witten/Herdecke University, 58455 Witten, Germany

**Keywords:** special care dentistry, dental education, postgraduate training, disability, psycho-emotional disorders, dental team

## Abstract

Background: The treatment of children and adolescents with disabilities (CA-Dis) and psycho-emotional disorders (CA-Psy) places special demands on dentists. Aim: To explore German dentists’ (with key expertise in paediatric dentistry) perception of their competence and comfort levels in dealing with these patients, and implications for access to care. Methods: Online questionnaire surveying demographic information and self-assessment of training, knowledge, and comfort in dealing with CA-Dis and CA-Psy among 1725 members of the German Society of Paediatric Dentistry (DGKiZ). Results: Ninety-two participants (11 male, 81 female) completed the questionnaire: 17.4% (*n* = 16) treated CA-Dis once or more a day; CA-Psy were rarely treated on a daily basis (7.6%; *n* = 7). In regard to CA-Dis, 62% (*n* = 57) rated their level of expertise as “good” or “very good”; for CA-Psy this was 40.2% (*n* = 37). Overall, 76.1% (*n* = 70), respectively, 88.0% (*n* = 81) of the respondents felt they had been inadequately prepared to treat CA-Dis or CA-Psy. Although the physical burden of treating CA-Psy was rated as “not at all stressful” or only “slightly stressful” by 45.7% of the participants, 31.5% rated the psychological distress as “very stressful” or “extremely stressful”. The better their self-assessed expertise in treating CA-Dis was, the lower their own psychological distress was rated (*r* = −0.34). Training on this topic seems to have an impact on the perceived burden of treating such patients. Conclusions: A core curriculum in special care dentistry needs to be embedded in the German dental curriculum. The results permit the development of health programs for workplace health management in dentistry.

## 1. Introduction

Statistically seen, every eighth person in Germany has a disability [1]. This insight rests on the number of approximately 10 million persons with disability living in Germany at the end of 2019, of which 7.9 million were even considered to have a severe disability [1]. Moreover, according to these data published by the German Federal Statistical Office, 194,213 of the persons with severe disability were aged between 0–17 years. In proportional terms, this means that 2.5% of the individuals with severe disability in Germany were children or adolescents at the time of the count. Furthermore, this statistical data illustrates that not all disabilities are congenital. Similarly, psycho-emotional disorders do not necessarily always exist from birth, even if they may often have genetic roots [2]. Overall, it appears that psycho-emotional disorders are affected by a variety of interacting factors. In Germany, the measured prevalence of psycho-emotional disorders is published to be between 17% and 28% in children and adolescents [3,4].

In contrast to the situation in paediatric and adolescent medicine in Germany, where interdisciplinary care is provided in specialized paediatric centres for developmental problems, for example, for children born with disabilities, similar care structures are typically not present for dentistry. In other words, in Germany, any dentist may find herself or himself in the position of having to treat a child or adolescent with a disability or a psycho-emotional disorder.

Across all age ranges, individuals in both of these patient subgroups are found to have poorer dental health than individuals of the same age in the general population. This insight is derived from national and international data on the dental and oral health of persons with disabilities or psycho-emotional disorders [5,6,7,8,9,10]. These data illustrate that, worldwide, stakeholders in health care systems need to view children and adolescents with disabilities or psycho-emotional disorders as particularly vulnerable groups of patients that may experience barriers to dental care. Specific care concepts are, therefore, needed for children and adolescents in these groups to improve equity of care. In this context, it is important to understand not only how well dentists with key expertise in paediatric dentistry consider themselves to be trained for the provision of dental care for clientele from these two groups, but also how they, themselves, experience the treatment and care of patients from these groups. 

Looking at the main aim and secondary aims of the study, it has to be taken into account that they refer only to the provision of dental care to children with disability (CA-Dis) or psycho-emotional disorders (CA-Psy) by dentists with key expertise in paediatric dentistry and working in Germany. The main aim of the present study was to explore how dentists perceive their own levels of expertise obtained in undergraduate and continuing education in regard to these special patient groups. Apart from this, several secondary aims were established: (A) Is provision of dental care for these patients perceived by the dentists as psychological distress and/or physical burden? (B) Does dentists’ knowledge as well as perceived distress and/or burden differ between patients with disability and patients with psycho-emotional disorders? (C) Is there a correlation between special knowledge of the dentists and the level of psychological distress and physical discomfort these dentists experience while treating these special patients? (D) Are the results of the present study comparable with those from self-assessment studies for dentists from two German federal states (Thüringen—central Germany, and Baden-Württemberg—southwestern Germany), which had been conducted approximately 10 years previously [11,12]. 

## 2. Materials and Methods

This observational questionnaire-based, cross-sectional study was conducted in collaboration with the German Society for Paediatric Dentistry, (*Deutsche Gesellschaft für Kinderzahnheilkunde e.V*.-DGKiZ). The DGKiZ, with headquarters in Würzburg (Bayern, Germany), is a nationwide, non-profit professional and academic association. Its members are dentists who aim to provide local dental services for children and their parents, within their home districts, as well as specialist care for chronically ill children or adolescents, or for those with disabilities. The surveyed participants were, therefore, German dentists whose key expertise is the treatment of children and adolescents. In this place, it should be pointed out that, in contrast to other countries, there is no officially recognized specialization in paediatric dentistry in Germany. It was, thus, only through the willingness of the members and board of the German Society for Paediatric Dentistry to collaborate with us on this study that we could reach a comparatively large collective of dentists with key expertise in paediatric dentistry for this survey.

***Online Questionnaire:*** A questionnaire with 39 (34 closed-ended and 5 open-ended) questions was specifically designed to collect the data for the study. Although fundamental study parameters, such as type, implementation, and scope of the survey, had been agreed on with contact partners from the dental society prior to the survey, the society had no influence over the content of the questionnaire. The questionnaire was consciously designed to be similar to those used in previous national and international studies to allow comparison of the results [11,12,13,14,15]. An add-on to the questionnaire was, however, included to shed light on the participants’ perception of stress and stress management skills. This add-on was in the form of a Stress Appraisal Measure (SAM) questionnaire [16]. Reis et al. described in detail the SAM questionnaire and checked the assumption of whether a dental examination of children and adolescents with autism spectrum disorders is viewed by these dentists as a challenge [17].

***Survey period and ethical aspects*:** This survey was conducted online using the online platform SoSci (SoSci Survey GmbH, München, Germany) between 18 August 2020 and 10 October 2020. Prior to conducting the survey, a positive vote had been obtained from the DGKiZ board, as well as written confirmation from the ethics committee of Witten/Herdecke University that no professional advice or ethical approval is needed for our survey because, in Germany, for anonymous surveys among employees in the healthcare sector such a vote is not required. In this context, we would also like to refer to a multicentre project that was methodologically based on the same regulations, and whose results have already been published [18]. Via its distribution list, the DGKiZ board sent e-mails containing the link to the questionnaire to its 1725 members with the invitation to participate in the online survey. Thus, no pre-selection, selection of a sample or target group of specialized DGKiZ members was defined, but all members were invited to participate. Four weeks after the survey had begun (21 September 2020), a reminder was sent via the same e-mail distribution list to guarantee that, in compliance with data privacy laws, the study group would not be able to deduce the respondents’ identities. The data were, hence, collected in compliance with the General European Data Protection Regulation. Before responding to the online questionnaire, the respondents had to indicate that their participation was voluntary. They also had to give their consent and confirm that they were at least 18 years old. Only fully completed questionnaires were included in the study.

***Transfer and Assessment of Data, and Statistics*:** For the evaluation of the survey results, the collected datasets were saved to a separate data storage device, which allowed authorized access only. The data were then processed using Excel 2016 (Microsoft Corp.; Redmond; Washington, DC, USA) and statistically analysed using SPSS Version 25 (IBM Corporation; New York, NY, USA). Parameters such as minimum value, maximum value, mean value, and standard deviation were determined for the descriptive statistics. In addition, the cross-sectional correlations were also calculated, after testing for normal distribution (Shapiro–Wilk Test).

## 3. Results

During the survey period, the online questionnaire was clicked, or called up, 342 times. In total, 192 respondents had begun answering the questionnaire, but, in the end, only 47.9% of these (*n* = 92; 11 male and 81 female) completed the entire questionnaire. The return or response rate in relation to all 1725 e-mails that had been sent out to members of the DGKiZ was thus 11.1% for all returned questionnaires, and 5.3% for the fully completed questionnaires included in this study. Originally, 96 completed questionnaires had been returned; however, four of these questionnaires were excluded from the analysis because the answers were incomplete. These four questionnaires were manually removed and were not included in the evaluation. In the following, the data are based on the responses of the 92 included participants.

***Sociodemographic Data:*** As depicted in Table 1, most respondents were aged between 35–54 years (*n* = 57; 62.0%). In questions that allowed multiple answers, most of the surveyed dentists (42.4%) responded that they either worked in their own dental practice or were dentists (*n* = 27; 29.3%) or assistant dentists (*n* = 2; 2.2%) employed in private practices. The description “private practice” means that the practice is engaged in the care of patients with statutory and/or private health insurance. In addition, the participants responded that a majority of their patients were statutorily insured. Three-quarters of the participating dentists (*n* = 69; 75%) responded that their dental practice or workplace was located in an urban area.

Table 1 also shows how the respondents assessed the wheelchair accessibility and wheelchair compatibility of the dental practice, as well as the frequency in which children and adolescents with disabilities (CA-Dis) and psycho-emotional disorders (CA-Psy) were treated in their dental practices.

**Figure 1:**
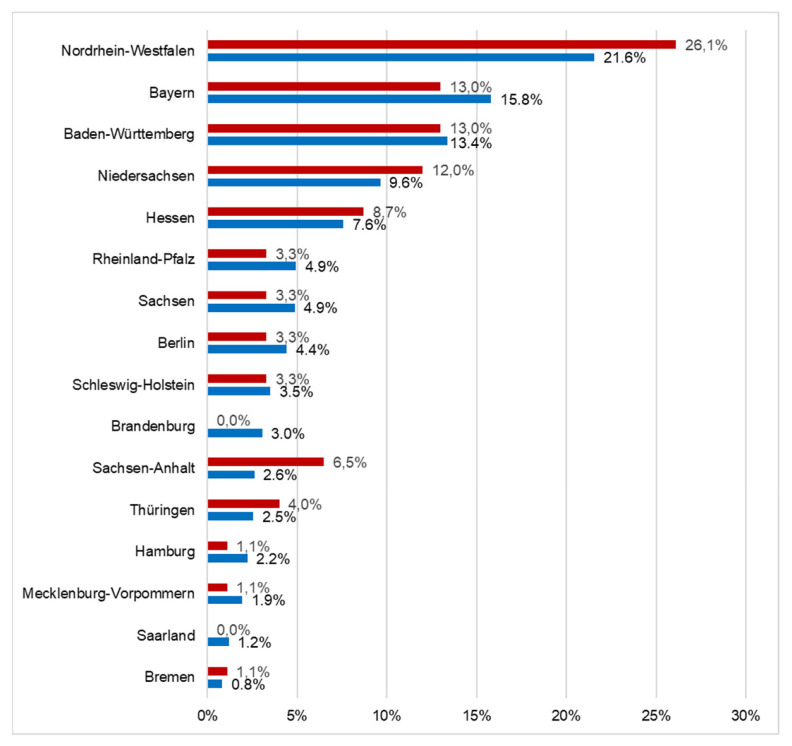
Distribution of the study participants (above—red) and the German population (below—blue) according to German federal states.

Further demographic details for the participants show that most had over 20 years of professional experience (*n* = 41; 44.6%); 24 of the participants had between 11–20 years of professional experience (26.1%), and 27 participants (29.3%) had up to ten years of professional experience. Moreover, the participants came from practically every German federal state. Figure 1 shows, that percentage distribution of the study participants largely corresponds to the percentage distribution of the German population (83.16 million) [19] by the 16 German federal states. 

***Self-assessment of dentists in regard to expertise, dental school education, and burden in treating CA-Dis and CA-Psy:*Table 2** presents an overview of how the responding dentists evaluated their own expertise, university dental education, psychological distress and physical burden in regard to treating CA-Dis and CA-Psy. The responses to these questions were on Likert scales. 

Table 2 also shows how the responding dentists rated their own expertise in treating CA-Dis und CA-Psy and the dentists’ outside assessment of their entire dental team’s expertise in treating and managing CA-Dis and CA-Psy.

The results in regard to the level of psychological distress and physical burden experienced by the responding dentists while treating CA-Dis or CA-Psy differed somewhat for the two patient groups: (1) In regard to treating CA-Dis, the respondents had, in almost equal proportions, replied the treatment was “not at all stressful” or only “slightly stressful” in terms of psychological distress (*n* = 39; 42.4%), respectively, in terms of physical burden (*n* = 36; 39.1%). (2) While a considerably large number of respondents indicated that they felt little or no physical burden (*n* = 42; 45.7) in treating CA-Psy, the psychological burden, on the other hand, was rated as “very stressful” or even “extremely stressful” by some (*n* = 29; 31.5%). Additionally, Table 2 also shows how the respondents experienced the patients’ willingness to cooperate, in the two patient groups.

***Correlation between special knowledge, dental education, and stress burden in dentists:*** The correlation between the perceived quality of the special knowledge taught during their undergraduate education at the university and postgraduate expertise and the degree of physical burden and psychological distress experienced by dentists while treating CA-Dis and CA-Psy is presented in Table 3. Because the data for the relevant variables did not have normal distribution (Shapiro–Wilk-Test), the significance of the correlations was not tested.

However, the better the dentists rated their own expertise in treating CA-Dis, the lower their self-reported psychological distress was while treating these patients (*r* = −0.34). A negative correlation, although lower than for CA-Dis, was also found between expertise level and self-reported psychological distress while treating CA-Psy (*r* = −0.16). Similarly, the better dentists rated their own expertise in treating CA-Dis, the lower they rated their own physical burden (*r* = −0.14). No notable correlation was found between expertise level and reported physical stress in the treatment of CA-Psy (*r* = 0.06).

The correlation between special knowledge acquired in dental school and the psychological distress and physical burden in treating both of these patients’ groups was mostly in the range of <10, and was thus inconclusive. 

***Special knowledge in regard to CA-Dis and CA-Psy acquired in postgraduate training courses for dentists:*** The following responses were found for the question “Have you attended one or more postgraduate training courses on the treatment of the patient groups relevant to this questionnaire?” (CA-Dis, CA-Psy, as well as children and adolescents with autism spectrum disorders): 58 participants answered “yes”; 34 participants answered “no”. In the space for free-form responses included for the answer “yes”, approximately half of the respondents noted that they had attended postgraduate training courses on the treatment of children and adolescents (children and adolescents with autism spectrum disorders, CA-Dis, CA-Psy). The most frequent free response (n = 23; 44.2%) was that postgraduate courses on autism spectrum disorders had been attended.

**Figure 2:**
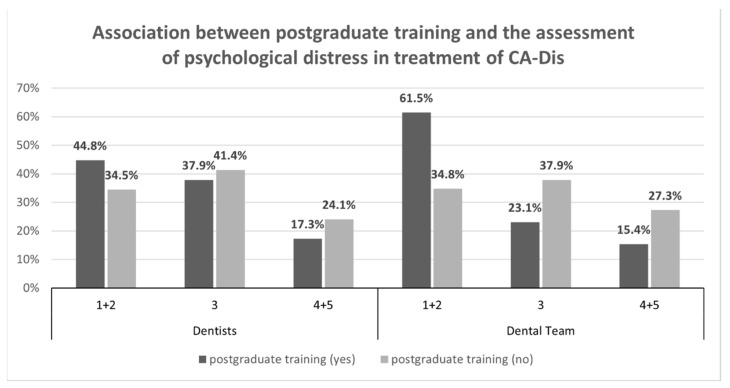
Association between postgraduate training and the assessment of psychological distress in the treatment of children and adolescents with disability (CA-Dis) (Abbreviations and captions: The answers were given on a scale from 1 = not at all stressful, 2 = slightly stressful, 3 = moderately stressful, 4 = very stressful, 5 = extremely stressful; CA-Dis = Children and adolescents with disabilities).

***Association between special knowledge from postgraduate training courses and stress burden of dentists:*** Overall, dentists who had indicated that they had attended advanced training courses answered more frequently that their stress burden while treating CA-Dis and CA-Psy was “not at all stressful” or only “slightly stressful” than did those who had not attended such advanced training courses. The answers “very stressful” and “extremely stressful” proved difficult to differentiate, particularly, since “extremely stressful” was chosen by only a few respondents (Figure 2).

***Dentists’ assessments of their dental teams in regard to special knowledge, management skills, and experienced burden in treating CA-Dis and CA-Psy:*** The correlations between the dental teams’ management skills and degree of special knowledge in treating CA-Dis und CA-Psy and the teams’ perceived levels of psychological and physical stress in treating these patient groups is presented in Table 4. Because the data for the relevant variables did not show normal distribution (Shapiro–Wilk-Test), the significance of the correlations was not tested. A high positive correlation was found between high levels of special knowledge about the special needs of CA-Dis in the dental teams and the perceived quality of management of these patients (*r* = 0.73). A similarly high positive correlation was also found in this regard for CA-Psy (*r* = 0.68). A positive correlation was also found between a good level of special knowledge in the teams in regard to CA-Psy and the quality of management in respect to both patient groups (CA-Dis: *r* = 0.65; CA-Psy: *r* = 0.78). The assessment of the dental team in regard to the management of CA-Dis and CA-Psy and the experienced physical burden and psychological distress were negatively correlated, but not strongly so. There was a stronger negative correlation between the teams’ special knowledge on CA-Dis and the psychological distress experienced while treating these patients (*r* = −0.21). The same was true for CA-Psy (*r* = −0.19). Although to a lesser degree, negative correlations were also found for all other relationships.

***Special knowledge in regard to CA-Dis and CA-Psy acquired in continuing education courses for dental teams:*** In response to the question “Have members of your dental team attended a continuing education course on treating the patient groups relevant to this survey?” 26 respondents replied “yes”, and 66 respondents replied “no”. Approximately half of the free-text responses on this topic stated that continuing education course on treating children and adolescents had been attended by team members (see above). Continuing education courses that were named in this context were “paediatric dentistry” and “special needs dentistry” (each *n* = 8; 30.8%), as well as “autism spectrum disorders” (*n* = 6; 23.1%).

***Association between special knowledge from continuing education courses and stress burden of dental teams:*** The majority of the dentists whose teams had not attended such training courses tended to consider treatment of CA-Dis and CA-Psy to be more in the stressful range for their teams, both in psychological and physical terms (Figure 2). The other responses in this section were inconclusive.

## 4. Discussion

The present study is the first to provide insights into how dentists in Germany who are members of the DGKiZ, and whose key expertise is the treatment and care of children and adolescents, assess their own and their dental teams’ level of expertise in the provision of care for children and adolescents with disabilities (CA-Dis) and psycho-emotional disorders (CA-Psy). 

A limitation of this study, which was the first of several of its kind (by this author´s group) to survey experts online, is that it was conducted only on members of the DGKiZ, a middle-sized academic society specific to paediatric dentistry and only one of several German associations in Dentistry. Possible approaches to conducting surveys of other professionals, e.g., German associations in Dentistry—and the expected low response rates—have been discussed elsewhere [17]. Although the response rate in our survey must be considered below average in terms of percentages, the actual number of responses (*n* = 92) is, however, comparable with that of a survey conducted in the United States among members of the Special Care Dentistry Association (*n* = 75) [15].

In our opinion, the present, nationwide survey results can, by and large, therefore, be considered representative for German dentists with key expertise in paediatric dentistry. This conclusion rests on the sociodemographic data, such as the gender ratio and regional distribution, by percentage (Figure 1) of the surveyed dentists. It must be explicitly stressed that this conclusion does not extend to all dentists in Germany. In a different online survey study that was conducted among DGKiZ members a few weeks after the present survey, similar sociodemographic data were also obtained, a finding that further underpins our conclusion that the results of the present survey may be considered representative for this group of German dentists [20].

One section of the survey concerned the scope of special knowledge in regard to treating and managing two subgroups of paediatric and adolescent patients—children and adolescents with disabilities (CA-Dis) and psycho-emotional disorders (CA-Psy). This choice of focus was guided by the existing literature on the topic: on the one hand, there are various articles that call attention to the fact that the treatment of persons with disabilities or psycho-emotional disorders may be challenging for the entire dental team [13,14]; on the other hand, there are also articles that point out that the treatment of patients with “special needs” requires a special set of knowledge and practical skills. The latter publications underscore the need for advanced dental training and education in this regard [15,21,22,23,24].

Through their own experience in working in special care dentistry, the two dental authors of this study know how taxing daily clinical work in this field can be. Dental teams can, for example, experience an increased physical burden in treating CA-Dis with restricted mobility who need to be treated in their wheelchairs. In addition, the behavioural particularities of children and adolescents with “special needs” are challenges with which the dental team need to deal [24]. Moreover, the daily work in this field is also determined by numerous administrative tasks that need to be fulfilled, such as scheduling appointments for patients requiring general anaesthesia or for out-of-office treatment of patients in residential facilities (visiting dental treatment). In this context, in cases with various uncommon disorders, syndromes, or conspicuous psychiatric behaviours that could have oral or dental relevance, there is also often a need for interdisciplinary cooperation and consultation, for example with paediatricians, paediatric neurologists, or child and adolescent psychiatrists. In view of this last aspect, the authors would again like to return to the paediatric centres for developmental problems referred to earlier in the text. An idea that merits consideration would be to include dental services in the structure of these units, as a further development in the scope of these ambulatory care centres. 

In our survey, two-thirds of the participants (62%) rated their own expertise in dealing with CA-Dis as “good” or “very good”. In regard to CA-Psy, the same applied for 40.1% of the participants. These findings are particularly interesting in view of the fact that the participants essentially already possessed a great deal of experience in dealing with children and adolescents without disability, as can be seen from the details on their professional experience: 70.7% of the respondents had more than 10 years of experience in the field, and two-thirds of the respondents even had more than 20 years of experience. Nonetheless, it appears that individuals from CA-Dis and CA-Psy population groups are still treated more rarely than children and adolescents in the general population. On the one hand, this conclusion is based on the finding that almost a quarter of the dentists responding to the questionnaire had not found the question on the frequency with which they treated these patient populations relevant and, on the other, that only a good third of the 70 dentists who had responded to the question about the frequency with which they treated these patient groups had responded that they treated children and adolescents from these groups, on average, at least once a week (Table 1).

A further insight from this study was that the majority of the surveyed dentists stated that they had been “inadequately” or only “poorly” instructed in dental school on how to treat patients from the relevant patient groups (76.1%—CA-Dis, 88.0%—CA-Psy). These responses are in accord with those from two regional surveys of German dentists that were conducted around 10 years ago [11,12]. In these previous two surveys, around 85% of the participating dentists had responded that they had been only poorly instructed on the treatment of persons with disability in dental school. Furthermore, on an international level, there have been numerous reports that dentists are inadequately educated and trained in dental school to treat patients with disabilities and special needs [13,14,22,23,25]. Although the responses from our survey of members of the DGKiZ in regard to their dental education at university revealed a less than satisfactory state of affairs, a few promising developments in regard to this topic can, nonetheless, be observed in Germany. For example, the first—and currently still only—chair for Special Care Dentistry in Germany was set up in 2015. With the inception of this chair, Germany joined the ranks of the countries around the world that have dental schools that hold a chair in special care dentistry [22]. In addition, the new regulation governing the acquisition of the license to practice dentistry in Germany, which came into force in 2021, for the first time permits inclusion of topics on the provision of dental care for special patients groups, e.g., patients with disabilities, as subject matter for the final state examination in dentistry [26]. Unfortunately, to date, the regulation still does not define minimum teaching standards for this subject. We, therefore, call for an expert panel to be set up to address this gap as soon as possible. In regard to minimum didactic content, the panel could be informed by the “Guidance for the core content of a Curriculum in Special Care Dentistry at the undergraduate level” of the International Association for Disability and Oral Health (iADH) or the contents of the curriculum taught by the existing German chair for Special Care Dentistry [21,27]. 

The necessity of drawing up a core curriculum in special care dentistry in Germany is further bolstered by the results of our study, which show that a solid foundation of special knowledge and training on treating CA-Dis and CA-Psy has a positive impact on the perceived psychological distress and physical burden experienced by dentists while treating these patients. Dentists who had attended postgraduate training courses on the topic (CA-Dis and CA-Psy) thus tended to experience less psychological distress and a lower physical burden while treating children or adolescents from these patient groups than did dentists who had not attended such courses (Figure 2). In this respect, our nationwide survey study confirms the results of a regional survey of dentists that was conducted in 2012 [12].

Because the dentists in our survey were also asked to answer questions related to the level of special knowledge and the stress burden of their entire dental team, the positive association between continuing education courses and lower stress levels can be seen to extend to the entire team (Figure 2). Although the responses in regard to the dental team represent an outside perception, as seen by the responding dentist, scientific surveys of assisting dental staff in Germany are, to date, rare. And very little is, therefore, known about the actual perceptions of assistant dental staff in regard to their expertise or psychological distress or physical burden, or in regard to their attitudes towards their daily work in German dental practices, regardless of whether these are general practices or practices with a focus on paediatric and adolescent dentistry [28,29]. The dental authors of this study are, hence, to the best of their knowledge, not aware of any previous German study that investigated the association between the acquisition of special knowledge and skills in the treatment of CA-Dis and CA-Psy and the psychological and physical burdens experienced by dental teams during treatment of these two patient groups.

Consequently, survey questions in regard to special knowledge ought to differentiate between two aspects in this sense: On the one hand, there should be questions about special knowledge acquired at dental school, and, on the other hand, there should be questions about special knowledge acquired in postgraduate training courses.

So, several observations emerged from our study: The first is that postgraduate training courses on the treatment of children and adolescents with disabilities (CA-Dis) and psycho-emotional disorders (CA-Psy) are available and the majority of the dentists surveyed in our study also used these offers. The second observation is that advanced training courses reduce dentists’ perceived stress levels by increasing their personal expertise and skill levels. As such, our results could provide an important impulse for the development of custom-tailored advanced training courses as a means of workplace healthcare management in dentistry. This second observation, in particular, is one that merits further investigation: if dental teams become aware that the acquisition of special knowledge about these patient groups can reduce their perceived stress burden in dealing with these patients, such training courses might help increase the frequency with which CA-Dis and CA-Psy receive dental care. Possibly, such training courses could, in this way, help lower the barriers to dental care for these patient groups.

## 5. Conclusions

This study:

- reveals that special knowledge on the treatment of CA-Dis and CA-Psy is inadequately taught in undergraduate education in German dental schools and a core curriculum in special care dentistry is needed;
- illustrates that continuing education courses on CA-Dis and CA-Psy help reduce the psychological and physical burden on the entire dental team when treating these patients;
- provides impulses for the development of training programs for workplace health management in dentistry, also with the aim of lowering the barriers to care for patients with special needs.

## Figures and Tables

**Table 1:** Characteristics of the study participants (dentists being members of the DGKiZ), who completed the questionnaires.

Background Characteristics of the Study Participants	Frequencies *n* = 92	
**Gender**		
Male	11	12.0%
Female	81	88.0%
**Age (in age groups)**		
**<35 years**	23	25.0%
<35 years/male	4	4.4%
<35 years/female	19	20.6%
**35–44 years**	21	22.8%
35–44 years/male	1	1.1%
35–44 years/female	20	21.7%
**45–54 years**	36	39.2%
45–54 years/male	4	4.4%
45–54 years/female	32	34.8%
**55–64 years**	8	8.7%
55–64 years/male	1	1.1%
55–64 years/female	7	7.6%
**≥65 years**	4	4.3%
≥65 years/male	1	1.1%
≥65 years/female	3	3.2%
**Working arrangement**		
Alone in his/her own practice	39	42.4%
Employed in a private practice as a dentist	27	29.3%
Employed in a private practice as an assistant dentist	2	2.2%
Employed in a dental school (university)	10	10.9%
Employed in a medical care centre	12	13.0%
Employed in a hospital or clinic at the university	3 (1,2)	3.3% (1.1%, 2.2%)
others (e.g., students, pensioners)	4	4.3%
**Practice location**		
urban area/town or city	69	75.0%
rural area	23	25.0%
**Health insurance of patient**		
Statutory insurance	70.0% to 95.0%	
Private insurance	5.0% to 30.0%	
**Practice characteristics**		
General dental practice without specialization	11	12.0%
General dental practice with a concentration on paediatric dentistry	20	21.7%
General dental practice with multiple concentrations, including paediatric dentistry	16	17.4%
General dental practice with multiple concentrations, including paediatric dentistry and special care dentistry	4	4.3%
General dental practice with a special paediatric division	8	8.7%
Dental practice specialized exclusively on paediatric and adolescent dentistry	22	23.9%
Unit of a university dental clinic	11	12.0%
**Wheelchair accessibility of practice**		
Rooms of the practice or workplace are wheelchair accessible for children	67	72.8%
Rooms of the practice or workplace are furnished to be wheelchair accessible for children	48	52.2%
**Treatment frequency of the patients (*n* = 70)**		
CA-Dis—once or more a day	16	17.4%
CA-Psy—once or more a day	7	7.6%
CA-Dis—once or more a week	15	16.3%
CA-Psy—once or more a week	17	18.5%
CA-Dis—once or more a month	28	30.5%
CA-Psy—once or more a month	25	27.2%
CA-Dis—once a year or never	11	12.0%
CA-Psy—once a year or never	21	22.8%

Abbreviations: CA-Psy = Children and adolescents with psycho-emotional disorders; CA-Dis = Children and adolescents with disabilities.

**Table 2:** Knowledge of the dentists and the dental team as well as perception of the psychological and physical burden.

Professional Attitudes	1 & 2	3	4 & 5	*M (SD)*
**Knowledge of the dentists ^†^**				
How would you rate your expertise in treating CA-Dis?	13.0% (12)	25.0% (23)	62.0% (57)	3.64 (±0.97)
How would you rate your expertise in treating CA-Psy?	22.9% (21)	37.0% (34)	40.1% (37)	3.18 (±1.14)
Was the instruction on the treatment of CA-Dis in dental school adequate?	76.1% (70)	15.2% (14)	8.7% (8)	1.72 (±1.05)
Was the instruction on the treatment of CA-Psy in dental school adequate?	88.0% (81)	5.4% (5)	6.6% (6)	1.47 (±0.91)
**Knowledge of the dental team ^†,‡^**				
How would you rate the knowledge/special knowledge of your dental team in regard to the special challenges in treating CA-Dis? ^†^	33.6% (31)	20.7% (19)	45.7% (42)	3.17 (±1.29)
How would you rate the knowledge/special knowledge of your dental team in regard to the special challenges in treating CA-Psy? ^†^	41.3% (38)	20.7% (19)	38.0% (6)	2.85 (±1.39)
How would you rate your dental team in regard to how they deal with the special challenges of treating CA-Dis? ^‡^	26.6% (6)	29.3% (27)	64.1% (59)	3.80 (±0.94)
How would you rate your dental team in regard to how they deal with the special challenges of treating CA-Psy? ^‡^	13.0% (12)	34.8% (32)	52.2% (48)	3.55 (±0.99)
**Psychological distress and physical burden of dentists ^§^**				
How would you rate your psychological distress while treating CA-Dis?	42.4% (39)	33.7% (31)	23.9% (22)	2.71 (±1.09)
How would you rate your psychological distress while treating CA-Psy?	31.5% (29)	37.0% (34)	31.5% (29)	2.96 (±1.05)
How would you rate your physical burden while treating CA-Dis?	39.1% (36)	34.8% (32)	26.1% (24)	2.78 (±1.10)
How would you rate your physical burden while treating CA-Psy?	45.7% (42)	32.6% (30)	21.7% (20)	2.67 (±1.08)
**Assessment of patient’s cooperation ^¶^**				
How would you rate the willingness of CA-Dis to cooperate during dental treatment in your daily clinical work?	23.9% (22)	56.5% (52)	19.6% (18)	2.90 (±0.81)
How do you rate the willingness of CA-Psy to cooperate during dental treatment in your daily clinical work?	27.2% (25)	62.0% (57)	10.9% (10)	2.82 (±0.64)

**^†^** The answers were given on a scale from 1 = inadequate, 2 = poor, 3 = fair, 4 = good, 5 = very good. ^‡^ The answers were given on a scale from 1 = very poor, 2 = poor, 3 = fair, 4 = good, 5 = very good. ^§^ The answers were given on a scale from 1 = not at all stressful, 2 = slightly stressful, 3 = moderately stressful, 4 = very stressful, 5 = extremely stressful. ^¶^ The answers were given on a scale from 1 = very poor, 2 = poor, 3 = fair, 4 = good, 5 = very good. Abbreviations: CA-Psy = Children and adolescents with psycho-emotional disorders; CA-Dis = Children and adolescents with disabilities; M = Mean value; SD = Standard deviation.

**Table 3:** Correlations for dentists’ special knowledge, dental education and burden in treating CA-Dis und CA-Psy.

	Special Knowledge: CA-Dis	Special Knowledge: CA-Psy	Dental Education: CA-Psy	Dental Education: CA-Dis	Psychological Distress: CA-Dis	Psychological Distress: CA-Psy	Physical Burden: CA-Dis	Physical Burden: CA-Psy
**Special knowledge: CA-Dis**		0.73 *	0.08	0.29	−0.34	−0.19	−0.14	−0.16
**Special knowledge: CA-Psy**			0.32	0.26	−0.12	−0.16	0.06	0.06
**Dental education: CA-Psy**				0.69	0.09	−0.08	0.10	0.03
**Dental education: CA-Dis**					−0.09	0.01	0.12	0.02
**Psychological** **distress:** **CA-Dis**						0.73	0.60	0.57
**Psychological** **distress:** **CA-Psy**							0.60	0.62
**Physical burden:** **CA-Dis**								0.79
**Physical burden:** **CA-Psy**								

Captions and abbreviations: *n* = 92; CA-Psy = Children and adolescents with psycho-emotional disorders; CA-Dis = Children and adolescents with disabilities. * Pearson’s correlation coefficient (r).

**Table 4:** Correlations for assessment of dental teams’ management skills, special knowledge, and burden in treating CA-Dis und CA-Psy.

	Assessment of Team Management: CA-Dis	Assessment of Team Management: CA-Psy	Team Special Knowledge: CA-Dis	Team Special Knowledge: CA-Psy	Own Psychological Distress: CA-Dis	Own Psychological Distress: CA-Psy	Own Physical Burden: CA-Dis	Own Physical Burden: CA-Psy
**Assessment of** **team management:** **CA-Dis**		0.80 *	0.73	0.65	−0.17	−0.08	−0.05	−0.13
**Assessment of** **team management:** **CA-Psy**			0.68	0.78	−0.08	−0.19	−0.03	−0.08
**Team special knowledge: CA-Dis**				0.89	−0.21	−0.12	−0.09	−0.13
**Team special knowledge: CA-Psy**					−0.16	−0.19	−0.07	−0.09
**Own psychological distress: CA-Dis**						0.73	0.60	0.57
**Own psychological distress: CA-Psy**							0.60	0.62
**Own physical** **burden: CA-Dis**								0.79
**Own physical** **burden: CA-Psy**								

Captions and abbreviations: *n* = 92; CA-Psy = Children and adolescents with psycho-emotional disorders; CA-Dis = Children and adolescents with disabilities, * *r* = Pearson’s correlation coefficient.

## Data Availability

Due to the strict European General Data Protection Regulation and the statement in the questionnaire to the study participants, no pseudonymised data will be passed on to third parties. The dataset generated from this study cannot be deposited in a public repository, because the study participant consent did not include data sharing permissions. A request for access to data for researchers who meet criteria for access to confidential data must be made to the senior author: Peter Schmidt, email: peter.schmidt@uni-wh.de, or to a representative of our Department of Special Care Dentistry, Dental School, Faculty of Health, Witten/Herdecke University, Germany and the board of the German Society of Paediatric Dentistry, Würzburg, Germany, too. Applicants wanting access to the dataset on which the analyses were performed must be prepared to conform to German privacy regulations. For further details, please contact, e.g., the data protection officer at the Witten/Herdecke University, Germany.
